# CD44^+^/CD24^-^ phenotype contributes to malignant relapse following surgical resection and chemotherapy in patients with invasive ductal carcinoma

**DOI:** 10.1186/1756-9966-31-59

**Published:** 2012-07-04

**Authors:** Yan Lin, Ying Zhong, Heng Guan, Xiaohui Zhang, Qiang Sun

**Affiliations:** 1 Department of Breast Disease, Peking Union Medical College Hospital, Peking Union Medical College, 1 Shuaifuyuan, Wangfujing, Beijing, 100730, China; 2 Department of Vascular Surgery, Peking Union Medical College Hospital, Peking Union Medical College, Beijing, 100730, China

## Abstract

**Background:**

Invasive ductal carcinoma is the most common type of breast malignancy, with varying molecular features and resistance to treatment. Although CD44+/CD24- cells are believed to act as breast cancer stem cells and to be linked to poor prognosis in some patients, the association between these cells and tumor recurrence or metastasis in all or some types of invasive ductal carcinoma is unclear.

**Methods:**

A total of 147 randomly selected primary and secondary invasive ductal carcinoma samples were assayed for expression of CD44, CD24, ER, PR, and Her2. The association between the proportions of CD44+/CD24- tumor cells and the clinico-pathological features of these patients was evaluated.

**Results:**

CD44+/CD24- tumor cells were detected in 70.1% of the tumors, with a median proportion of 5.8%. The proportion of CD44+/CD24- tumor cells was significantly associated with lymph node involvement (*P* = 0.026) and PR status (*P* = 0.038), and was correlated with strong PR status in patients with recurrent or metastatic tumors (*P* = 0.046) and with basal-like features (*p* = 0.05). The median disease-free survival (DFS) of patients with and without CD44^+^/CD24^-/low^ tumor cells were 22.9 ± 2.2 months and 35.9 ± 3.8 months, and the median overall survival (OS) of patients with and without CD44^+^/CD24^-/low^ tumor cells were 39.3 ± 2.6 months and 54.0 ± 3.5 months, respectively, and with both univariate and multivariate analyses showing that the proportion of CD44^+^/CD24^-/low^ tumor cells was strongly correlated with DFS and OS.

**Conclusion:**

The prevalence of CD44+/CD24- tumor cells varied greatly in invasive ductal carcinomas, with the occurrence of this phenotype high in primary tumors with high PR status and in secondary tumors. Moreover, this phenotype was significantly associated with shorter cumulative DFS and OS. Thus, the CD44^+^/CD24^-^ phenotype may be an important factor for malignant relapse following surgical resection and chemotherapy in patients with invasive ductal carcinoma.

## Introduction

Invasive ductal carcinoma is the most common breast malignancy and a leading cause of cancer-related death in women worldwide.[1] Despite developments in surgical methods, cytotoxic chemotherapy, and agents targeted against estrogen receptor (ER) and HER2, a subset of patients with advanced stage invasive ductal carcinoma may experience tumor recurrence or metastasis within several years after treatment. It has been estimated that 11% of women with invasive ductal carcinoma will experience a recurrence within five years after surgery, including 8% of women with luminal A breast cancers and 15% of women with tumors having basal-like features.[2,3] The cancer stem cell hypothesis was proposed to explore breast cancer heterogeneity and the risk of breast cancer recurrence, and these cell subpopulations may contribute to drug resistance that drives tumor recurrence or metastasis [4].

Using keratin profiling, Hoechst dye efflux, and flow cytometry analysis of cell surface markers such as CD44, CD24, CD133, epithelial cell adhesion molecule, and mucin-1,[5] normal human breast stem-cell like cells have been independently identified as showing elevated expression of CD44 and no expression of CD24 (CD44+/CD24-), as well as elevated levels of stem cell enriched genes.[6] The CD44+/CD24- subpopulation was believed to be putative stem cells in human breast tissue, enriched for basal cells and motility genes, which could be generated during the epithelial-mesenchymal transition. Moreover, these cells were negative for mucin 1, estrogen receptor (ER), and v-erb-b2 erythroblastic leukemia viral oncogene homolog 2 (erbB2) receptor.[7,8] More importantly, high expression of CD44+/CD24- cancer cells was associated with poor patient prognosis.[9] These cells had the phenotype of cancer cells during the epithelial to mesenchymal transition, [10] indicating that the gene expression pattern of CD44+/CD24- cells in breast cancers resembled more closely the pattern observed in CD44+/CD24- cells in normal breast than that of CD44-/CD24+ cells isolated from the same tumor.[6] Taken together, these findings indicated that CD44+/CD24- cells, especially those expressing epithelial cell adhesion molecule, were breast cancer stem cells (CSCs).[11] In contrast, breast cancer cells expressing elevated levels of aldehyde dehydrogenase 1 (ALDH1) were also described as breast CSCs, with ALDH1+/CD44+/CD24- cells displaying strong tumorigenic potential.[12] Moreover, breast CSCs were believed to constitute up to 35% of the cancer cells in a tumor, whereas these cells constituted only about 1% of stem and progenitor cells present in normal breast [13].

Although CD44+/CD24- cells were regarded as breast CSCs and to be linked with poor prognosis in some breast cancer patients, the association between these cells and tumor recurrence or metastasis in all or some patients with breast cancer, especially those with invasive ductal carcinoma, has been unclear. Despite the fact that all intrinsic subtypes of breast cancer have the same CSCs, tumor relapse has been found to differ among patients with different intrinsic subtypes of invasive ductal carcinoma. Moreover, although CD44+/CD24- breast cancer cells have invasive properties, not all breast cancer cells with the CD44+/CD24- phenotype were able to grow as metastatic tumors whereas others showed aggressive metastatic growth.[14] In addition, although some primary tumors were predominantly CD44+, metastases at certain sites lacked any CD44 expression. [10] We therefore investigated whether breast cancer cells with the CD44+/CD24- phenotype are associated with the metastasis of different subtypes of invasive ductal carcinoma, and whether breast cancer CD44+/CD24- cells possess essential characteristics of cells with a metastatic phenotype.

## Materials and methods

### Patients and specimens

A total of 147 invasive ductal carcinoma samples were randomly selected from our tissue database. Patients had been treated at the Peking Union Medical College Hospital between April 2000 and December 2007. None of these patients had received neoadjuvant chemotherapy or radiotherapy. Clinical information was obtained by reviewing preoperative and perioperative medical records, or by telephone or written correspondence. Patients were staged based on the tumor-node-metastases (TNM) classification of the International Union Against Cancer, revised in 2002.[15] The use of these human materials in this study was approved by the Peking Union Medical College Hospital Medical Ethics Committee.

Patient clinical characteristics are shown in Table 1. Fresh-frozen tumor tissue samples were used for routine determination of estrogen receptor (ER), progestogen receptor (PR), and human epidermal growth factor receptor (Her2). Paraffin specimens of these tumors were collected and 5 mm thick tissue sections were cut and fixed onto silicified slides. Each sample was stained with hematoxylin and eosin (H&E) and histologically typed according to the World Health Organization (WHO) classification [16]. Tumor size and the number and location of metastatic lymph nodes were obtained from pathology reports. Basal-like features of tumor was defined as immunohistochemically negative for both SR and Her2.

**Table 1:** Demographic and clinical characteristics of patients with and without recurrence or metastasis

	**Without recurrence/metastasis**	**With recurrence/metastasis**	***P***
***N***	**56**	**91**	
Age (years)	50.8 ± 12.8 (13.0-77.0)	52.2 ± 12.4 (15.0-81.0)	0.510
Tumor size (cm)	3.2 ± 1.9 (1.2-9.5)	3.0 ± 1.6 (0.4-8.2)	0.437
Lymph node involvement	45 (80.4%)	70 (76.9%)	0.624
TNM stages			
I	5 (8.9%)	9 (9.9%)	0.511
II	24 (42.9%)	32 (35.2%)	
III	27 (48.2%)	49 (53.8%)	
IV	0 (0.0%)	1 (1.1%)	
ER expression	26 (46.4%)	31 (34.1%)	0.168
PR expression	28 (50.0%)	36 (39.6%)	0.266
Her2 expression	29 (51.8%)	41 (45.1%)	0.471
Basal-like feature^*^	9 (16.1%)	30 (33.0%)	0.018
Recurrence		40 (44.0%)	
Metastasis			
Skin		2 (2.2%)	
Lung		20 (22.0%)	
Liver		8 (8.8%)	
Bones		11 (12.1%)	
Brain		5 (5.5%)	
Others		5 (5.5%)	

ER, estrogen receptor; PR, progesterone receptor; Her2, human epidermal growth factor receptor 2; IDC, Invasive ductal carcinoma.

^*^ Immunohistochemically negative for both SR and Her2.

### Immunohistochemical staining and evaluation

Briefly, each tissue section was deparaffinized, rehydrated and incubated with fresh 3% hydrogen peroxide (H_2_O_2_) in methanol for 15 min. After rinsing with phosphate-buffered saline (PBS), the samples were immersed in 0.01 M sodium citrate buffer (pH 6.0) and heated in a microwave oven at 100 °C for 15 min for antigen retrieval. Non-specific binding was blocked by incubating the sections with normal goat serum for 15 min at room temperature. The samples were subsequently incubated at 4 °C overnight with different primary antibodies. The primary antibodies used included rabbit polyclonal antibody to CD44 (CD44v6, IgG, 1:50, Abcam, Cambridge, UK), mouse monoclonal to CD24 (IgG, 1:50, Thermo Electron Corp., Burlington, ON, CA), FITC linked mouse monoclonal antibody to SABC (1:50), and goat anti-rabbit Cy3 antibody (IgG, 1:20). CD44 was detected with permanent red and CD24 was detected with diaminobenzidine. ALDH1 was detected with a monoclonal rabbit anti-ALDH1 antibody (ALDH1A1, IgG, 1:100, Abcam) followed by EnVision™ on a Tech-Mate™ (DAKO). All slides were counterstained with hematoxylin to identify nuclei. All samples were scored twice by one person in a blinded fashion, with all unclear results discussed with a pathologist. If there were staining discrepancies among the three cores from the same patient, an average was used. CD44 staining was detected mainly in the membrane and CD24 staining was detected mainly in the cytoplasm. The proportion of CD44+/CD24- tumor cells was defined as the percentage of cells positive for permanent red staining but negative for diaminobenzidine staining. The results of CD44+/CD24- tumor cells proportion were classified into two groups, high and low, with a cut-off value based on the median value of their proportion.

### Statistical analysis

All calculations were performed using SPSS V.14.0 statistical software (Chicago, IL, USA). Associations between the presence of CD44, CD24 or different CD44/CD24 phenotypes and clinical variables as well as breast cancer subgroups were assessed by Fisher's exact test, except for age where the Mann–Whitney *U* test was used. Multivariate analysis was performed using Cox proportional hazards regression to determine the prognostic effect on disease-free survival (DFS) and overall survival (OS), and the log-rank test to compare survival between two strata. Hazard ratios (HR) and their corresponding 95% confidence intervals (CI) were computed to provide quantitative information about the relevance of the results of statistical analysis. All tests were two-sided and *P* < 0.05 was considered statistically significant.

## Results

### Patient characteristics

The baseline characteristics of the study population are given in Table 1. All patients were female, with a mean ± standard deviation (SD) age of 51.6 ± 12.5 years (range, 13.5 to 80.7 years) and a mean ± SD tumor size of 3.1 ± 1.8 cm (range, 0.4 to 9.5 cm). Lymph node involvement was positive in 115 patients (78.2%). According to TNM classification, 14 patients (9.5%) were stage I, 56 (38.1%) were stage II, 76 (51.7%) were stage III, and 1 (0.7%) was stage IV. Of the 147 patients, 57 (38.8%) were positive for ER expression, 64 (43.5%) were positive for PR, 70 (47.6%) were positive for Her2, and 39 (26.5%) were positive for basal-like features (defined as immunohistochemically negative for both SR and Her2). Of the 147 patients, 87 (59.2%) were received adjuvant chemotherapy and 95 (64.6%) were received agents targeted against estrogen receptor. Median follow-up time was 23.0 months (range, 2 to 91 months), during which 40 patients (27.2%) experienced tumor recurrence and 51 (34.7%) developed metastases.

### Presence of CD44+/CD24- phenotype in invasive ductal carcinoma tissue

**Figure 1:**
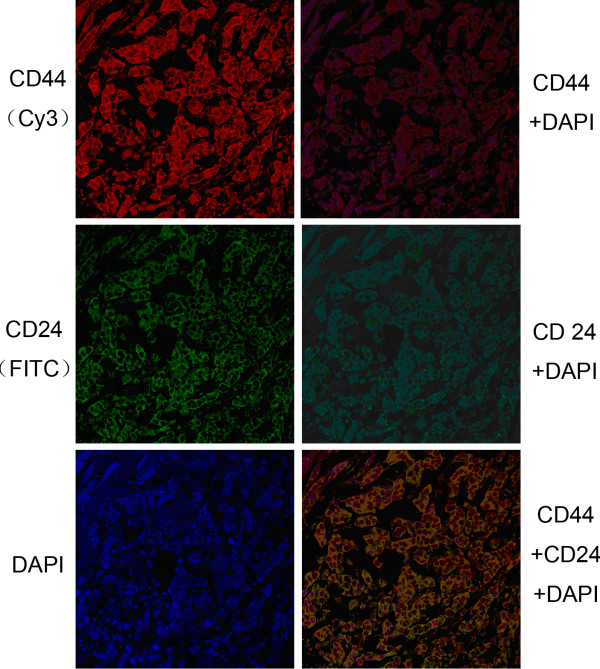
Immunohistochemical staining for CD44, CD24, and DAPI (×400).

The presence of CD44 and CD24 antigens on invasive ductal carcinoma tissues was analyzed using double-staining immunohistochemistry. Figure 1 displays representative staining patterns of CD44 and CD24. CD44 was visible primarily as membranous permanent red staining, with only eight tumors displaying cytoplasmic and membranous staining. CD24 was visible mainly as cytoplasmic diaminobenzidine staining, with only six tumors displaying membrane diaminobenzidine staining. To determine the proportion of tumorigenic CD44+/CD24- cells within each tumor, we scanned for the presence of permanent red staining without any diaminobenzidine interference. CD44+/CD24- tumor cells were present in 103 of the 147 (70.1%) tumors, but absent from the other 44 (29.9%), with the proportion of tumor cells expressing this phenotype ranging from a few to 70%, with a median proportion of 5.8%, and this median proportion was selected to categorize patients as CD44+/CD24- tumor cells high group and CD44+/CD24- tumor cells low group according to cutoff definition. The frequency of tumors with different proportions of CD44+/CD24- tumor cells is presented in Table 2. The proportions of CD44+/CD24- tumor cells in clinical specimens correlated significantly with lymph node involvement (*P* = 0.026) and PR expression (*P* = 0.038). Higher proportions of CD44+/CD24- tumor cells were observed in specimens from patients with (19.20%) than without (8.66%) lymph node involvement and with (21.06%) than without (13.09%) PR expression. There were no associations between percentage of CD44+/CD24- tumor cells and patient age, tumor size, TNM stages, ER or Her2 expression, and basal-like features.

**Table 2:** Proportion of all patients and patients with recurrence/metastasis and CD44/CD24 data with CD44^+^/CD24^-/low^ tumor cells

	***n***	**All cases (%)**	***P***	***n***	**Recurrence/metastatic cases (%)**	P*
Age (years)						
< 50	74	18.34 ± 2.70	0.444	34	24.91 ± 3.79	0.022
≥ 50	73	15.45 ± 2.66		38	13.20 ± 3.32	
Tumor size						
T1	47	15.78 ± 2.86	0.224	15	13.19 ± 3.53	
T2	76	20.12 ± 2.90		44	23.78 ± 3.68	
T3 + T4	17	10.27 ± 4.46		13	11.83 ± 6.60	0.152
Lymph node involvement						
Absent	32	8.66 ± 2.70	0.026	18	10.00 ± 3.77	0.075
Present	115	19.20 ± 2.29		54	21.53 ± 3.19	
TNM stage						
I + II	70	15.87 ± 2.63	0.500	33	16.88 ± 3.74	0.368
III + IV	77	18.49 ± 2.81		39	21.73 ± 3.79	
ER expression						
Negative	90	16.49 ± 2.47	0.845	47	18.92 ± 3.17	0.944
Positive	57	17.26 ± 3.07		25	19.32 ± 4.81	
PR expression						
Negative	83	13.09 ± 2.41	0.038	43	14.63 ± 3.06	0.046
Positive	64	21.06 ± 2.98		29	25.32 ± 4.51	
Her2 expression						
Negative	77	16.18 ± 3.03	0.566	38	17.36 ± 4.17	0.441
Positive	70	18.47 ± 2.61		34	21.57 ± 3.47	
Basal-like feature ^†^						
Absent	108	18.44 ± 2.24	0.143	49	11.70 ± 4.07	0.050
Present	39	11.93 ± 3.66		23	22.66 ± 3.30	
Recurrence or metastasis						
Absent	75	14.26 ± 2.72	0.246			
Present	72	18.73 ± 2.58				
Lesions in recurrence/metastatic patients						
Primary				56	15.39 ± 2.63	0.014
Secondary				16	30.41 ± 6.46	

* Calculated by t tests. ER, estrogen receptor; PR, progesterone receptor; Her2, human epidermal growth factor receptor 2.

^†^ Immunohistochemically negative for both SR and Her2.

### Association of CD44+/CD24- phenotype with steroid receptor status

Of the 121 samples with CD44/CD24 data, 56 (46.2%) were positive for PR expression. CD44+/CD24- status was significantly correlated with strong PR staining in all patients (*P* = 0.038) and in samples from patients with recurrence or metastasis (*P* = 0.046). Interestingly, although ER expression was observed in 50 of the 121 (41.3%) patients with CD44/CD24 data, the presence of CD44+/CD24- tumor cells was not significantly correlated with positive ER expression in all patients and in patients with recurrence or metastasis.

### Association of CD44+/CD24- phenotype with basal-like feature

We found that the proportion of CD44+/CD24- tumor cells was similar in breast cancer samples with and without basal-like features (11.93% versus 18.44%, *p* = 0.143). However, in samples from patients with tumor recurrence or metastasis, the proportion of CD44+/CD24- tumor cells was significantly higher in breast cancer tissue with basal-like features than in tissue without such features (22.66% versus 17.70%, *p* = 0.05).

### Association of CD44+/CD24- phenotype with DFS and OS: univariate analysis and multivariate analysis

**Figure 2:**
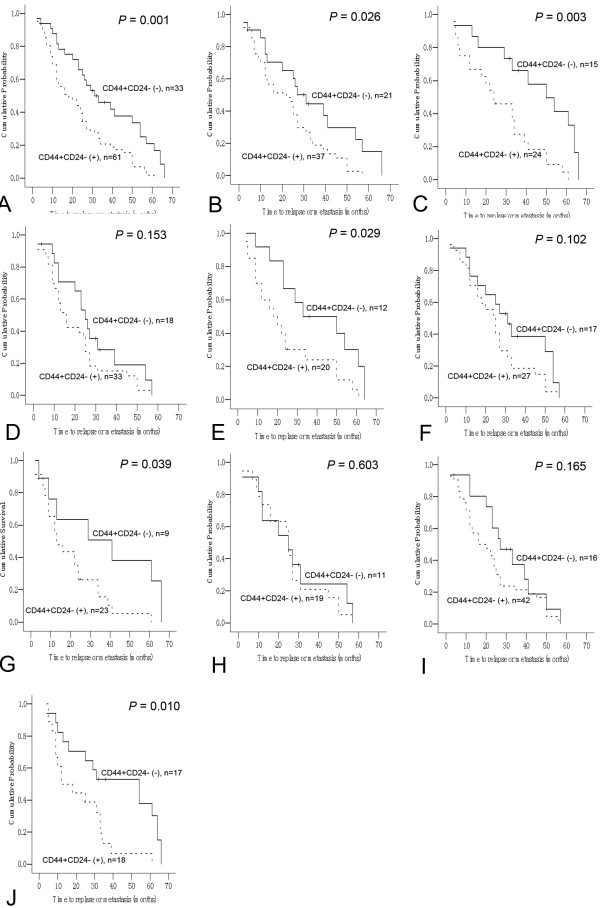
**Analysis of disease-free survival (DFS) in breast cancer patients with and without the CD44+/CD24- phenotype.A.** All patients; **B.** Patients with invasive ductal carcinoma; **C.** Progesterone receptor (PR) negative patients; **D.** PR positive patients; **E.** Estrogen receptor (ER) negative patients; **F.** ER positive patients; **G.** Her2 negative patients; **H.** Her2 positive patients; **I.** Patients with basal-like features; **J.** Patients not receiving postoperative immunotherapy; **K.** Patients receiving postoperative immunotherapy.

The results of univariate analyses of the associations between each individual predictor and DFS are shown in Table 3. The proportion of CD44^+^/CD24^-/low^ tumor cells (*P* = 0.002), PR status (*P* = 0.004), basal-like feature (*P* = 0.007), and TNM stage (*P* = 0.029) were strongly correlated with DFS. Kaplan-Meier analysis showed that the presence of CD44^+^/CD24^-/low^ tumor cells was significantly associated with shorter DFS compared with the absence of CD44^+^/CD24^-/low^ tumor cells (22.9 ± 2.2 months versus 35.9 ± 3.8 months; Pearson chi-square, 10.696, *p* = 0.001; Figure 2A). When all predictors were included in a Cox model (multivariate analysis, Table 3), the presence of CD44^+^/CD24^-/low^ tumor cells (hazard ratio, 1.931; *P* = 0.011), PR status, basal-like feature, and TNM stage retained their prognostic significance for DFS.

**Table 3:** Univariate and multivariate analyses of the relationship of CD44^+^/CD24^-/low^ tumor cells to disease-free survival

**Variable**	**Univariate analysis**			**Multivariate analysis**		
	**HR**	**95% CI**	***p*-value**	**HR**	**95% CI**	***p*-value**
CD44^+^/CD24^-/low^ tumor cells						
High	2.144	1.321-3.479	0.002	1.963	1.163-3.313	0.012
Low	1.000			1.000		
ER status						
Positive	0.826	0.524-1.304	0.412	1.425	0.731-2.776	0.298
Negative	1.000			1.000		
PR status						
Positive	0.500	0.312–0.800	0.004	0.192	0.088–0.420	0.001
Negative	1.000			1.000		
Her2 status						
Positive	0.966	0.614–1.521	0.882	0.692	0.317–1.513	0.357
Negative	1.000			1.000		
Basal-like feature*						
Present	2.731	0.461-1.393	0.007	3.902	1.402-10.859	0.009
Absent	1.000			1.000		
TNM stage						
Stage III/IV	1.989	0.814–2.626	0.029	1.820	1.051–3.151	0.033
Stage I/II	1.000			1.000		
Lymph node involvement						
Absent	0.724	0.427-1.227	0.230	1.081	0.540-2.164	0.827
Present	1.000			1.000		
Age (years)						
≥ 50	1.047	0.681–1.610	0.883	1.062	0.627–1.799	0.822
< 50	1.000			1.000		

Abbreviations: HR, hazard ratio estimated from Cox proportional hazard regression model; CI, confidence interval of the estimated HR. ER, estrogen receptor; PR, progesterone receptor; Her2, human epidermal growth factor receptor 2.

^*^ Immunohistochemically negative for both SR and Her2.

Meanwhile, the results of univariate analyses of the associations between each individual predictor and OS are shown in Table 4. Similarly with the relation with DFS, the proportion of CD44^+^/CD24^-/low^ tumor cells (*P* = 0.001), basal-like feature (*P* = 0.029), and TNM stage (*P* = 0.027) were strongly correlated with OS. Kaplan-Meier analysis showed that the presence of CD44^+^/CD24^-/low^ tumor cells was significantly associated with shorter OS compared with the absence of CD44^+^/CD24^-/low^ tumor cells (39.3 ± 2.6 months versus 54.0 ± 3.5 months; Pearson chi-square, 12.140, *p* = 0.001). When all predictors were included in a Cox model (multivariate analysis, Table 4), the presence of CD44^+^/CD24^-/low^ tumor cells (hazard ratio, 2.237; *P* = 0.002), basal-like feature, and TNM stage retained their prognostic significance for OS.

**Table 4:** Univariate and multivariate analyses of the relationship of CD44^+^/CD24^-/low^ tumor cells to overall survival

**Variable**	**Univariate analysis**			**Multivariate analysis**		
	**HR**	**95% CI**	***p*-value**	**HR**	**95% CI**	***p*-value**
CD44^+^/CD24^-/low^ tumor cells						
High	2.193	1.383-3.477	0.001	2.237	1.345-3.720	0.002
Low	1.000			1.000		
ER status						
Positive	0.757	0.488-1.175	0.215	1.164	0.585-2.314	0.665
Negative	1.000			1.000		
PR status						
Positive	0.702	0.457–1.078	0.106	0.968	0.496–1.888	0.924
Negative	1.000			1.000		
Her2 status						
Positive	0.932	0.605–1.435	0.748	1.583	0.782–3.201	0.201
Negative	1.000			1.000		
Basal-like feature*						
Present	0.608	0.389-0.949	0.029	0.342	0.131-0.891	0.028
Absent	1.000			1.000		
TNM stage						
Stage III/IV	1.614	1.055–2.470	0.027	1.652	1.014–2.690	0.044
Stage I/II	1.000			1.000		
Lymph node involvement						
Absent	0.891	0.528-1.504	0.666	0.674	0.343-1.323	0.251
Present	1.000			1.000		
Age (years)						
≥ 50	1.110	0.735–1.676	0.621	1.384	0.847–2.260	0.194
< 50	1.000			1.000		

Abbreviations: HR, hazard ratio estimated from Cox proportional hazard regression model; CI, confidence interval of the estimated HR. ER, estrogen receptor; PR, progesterone receptor; Her2, human epidermal growth factor receptor 2.

^*^ Immunohistochemically negative for both SR and Her2.

### Presence of CD44+/CD24- phenotype in secondary invasive ductal carcinoma

We separately analyzed the secondary lesions from 56 patients with invasive ductal carcinoma and metastasis or recurrence. We found that a significantly higher proportion of secondary than primary lesions were positive for CD44^+^/CD24^-/low^ tumor cells (26.9% versus 7.0%, *P* < 0.05).

## Discussion

Invasive ductal carcinoma is the most common breast malignancy in women, with relapse or metastasis frequently occurring after surgical resection. CD44^+^/CD24^-^ breast cancer cells have been reported to have tumor-initiating properties.[17,18] We therefore investigated the importance of this breast CSC phenotype in the relapse and metastasis of invasive ductal carcinoma cells. Breast CSCs have been reported to constitute up to 35% of cancer cells in a tumor, compared with approximately 1% of stem and progenitor cells present in normal breast. [13] However, the size of the CSC pool in breast cancers is unclear, since one study showed that CSCs constitute less than 10% of cells in 78% of breast tumors,[19] whereas another study found that CD44^+^/CD24^-^ cells were present in all breast cancer samples. We therefore determined the percentage of CD44^+^/CD24^-^ cells in tissue samples from 147 invasive ductal carcinomas. We found that the size of the CSC pool ranged from 0% to 70%, with a median of 5.8%, and that CSCs constituted less than 22% of the cells in 75% of primary tumors. In contrast, tumor cells with this phenotype have been reported in 31% of primary breast tumors. This discrepancy may be due to different subtypes of breast cancers and different percentages of samples from primary and metastatic breast tumors.

Although CD44+/CD24- percentage was not associated with ER or HER2 expression, we observed an association between high CD44+/CD24- percentage and PR expression. This linkage was more prominent in samples from recurrent and metastatic tumors with more than 25% CD44+/CD24- cells. In contrast, previous studies showed that the presence of CD44+/CD24- tumor cells was not associated with ER or PR status [20].

CD44+/CD24- cells have been observed in 63% of basal-like subtype (SR-HER2- basal-like) breast tumors.[20] Although we did not observe a significant difference in the proportion of CD44+/CD24- cells in samples from tumors with and without basal-like features, we found that the CD44+/CD24- subpopulation was higher in samples of recurrent and metastatic tumors with basal-like features. Several studies have shown an association between CD44+/CD24- cells and the metastasis of basal-like breast cancers. For example, the expression of several metastasis-associated genes was found to be higher in cells with than without the CD44+/CD24- phenotype, and only malignant cell lines with the CD44+/CD24- subpopulation were able to invade matrigel, indicating that CD44+/CD24- cancer cells are more metastatic than non-CD44+/CD24- cells [21,22].

Importantly, a unique 186-gene invasiveness gene signature has been observed in CD44+/CD24- malignant cells,[22] linking the presence of CD44+/CD24- cells to distant metastasis although not to survival.[8,23] We found that the time to tumor relapse (including recurrence and metastasis) was significantly shorter in patients with than without CD44+/CD24- tumor cells. Metastasis is a complex process involving invasion, intravasation, survival in the blood stream, extravasation and homing and proliferation at the sites of metastasis.[8,24,25] The poor prognosis of patients with primary tumors having higher levels of CD44+/CD24- cells, but whose metastatic cells had the CD44±/CD24+ phenotype,[26,27] suggests that CD44+/CD24- tumor cells may be a transient phenotype and that these cells have an intrinsic program to transition to a phenotype that enhances their heterotypic interaction and survival/proliferation in distant organs.[8] This hypothesis, however, cannot explain the difference in time to tumor relapse in patients with and without CD44+/CD24- cancer cells who had undergone surgical resection plus immunotherapy.

## Conclusion

We observed variations in the prevalence of CD44+/CD24- tumor cells in breast tumors of different subtypes. This phenotype was highly prevalent in primary tumors with high PR expression and in secondary tumors. These results provide further evidence for the biological heterogeneity of breast cancers and for the enrichment in putative tumor-initiating cells in tumors with high PR expression and in secondary tumors. Moreover, the presence of CD44^+^/CD24^-/low^ tumor cells was associated with a shorter cumulative DFS and OS, suggesting that the CD44^+^/CD24^-^ phenotype may be an important factor of malignant relapse in patients with surgically resected invasive ductal carcinoma after chemotherapy.

## Abbreviations

ALDH1, aldehyde dehydrogenase; CSC, cancer stem cell; DAB, 3,3'-diaminobenzidine; DAPI, 4,6-diamidino-2-phenylindole; DFS, disease free survival; EDTA, ethylene diamine tetraacetic acid; EFS, event free survival; EGFR, epidermal growth factor receptor; ER, estrogen receptor; FITC, fluorescein isothiocyanate; IQR, interquartile-range; HER-2, human epidermal growth factor receptor; IHC, immunohistochemistry; LN, lymph node; NTP, nucleoside triphosphate; PBS, phosphate buffered saline; PCR, polymerase chain reaction; PR, progestogen receptor; SP, side population; SR, steroid receptor; RNase, ribonuclease.

## Competing interests

The authors declare that they have no conflicts of interest. All work was performed at the Department of Breast Disease, Peking Union Medical College Hospital, Peking Union Medical College.

## Authors' contributions

YL and YZ participated in the design of the study, evaluated the immunostaining results, performed the statistical analysis and drafted the manuscript. HG supported the statistical analysis. XZ supported the evaluation of the immunohistochemical results. QS conceived of the study, participated in its design, and helped to draft the manuscript. All authors read and approved the final manuscript.
